# Macaque models of enhanced susceptibility to HIV

**DOI:** 10.1186/s12985-015-0320-6

**Published:** 2015-06-14

**Authors:** Tara R. Henning, Janet M. McNicholl, Sundaram A. Vishwanathan, Ellen N. Kersh

**Affiliations:** Division of HIV/AIDS Prevention, Centers for Disease Control and Prevention, 1600 Clifton Road NE, MS A-25, Atlanta, GA 30333 USA

**Keywords:** SIV, SHIV, HIV, Susceptibility, Risk, Nonhuman primate model, Coinfection model

## Abstract

There are few nonhuman primate models of enhanced HIV susceptibility. Such models can improve comprehension of HIV acquisition risk factors and provide rigorous testing platforms for preclinical prevention strategies. This paper reviews past, current, and proposed research on macaque HIV acquisition risk models and identifies areas where modeling is significantly lacking. We compare different experimental approaches and provide practical considerations for designing macaque susceptibility studies. Modifiable (mucosal and systemic coinfections, hormonal contraception, and rectal lubricants) and non-modifiable (hormonal fluctuations) risk factors are highlighted. Risk acquisition models via vaginal, rectal, and penile challenge routes are discussed. There is no consensus on the best statistical model for evaluating increased susceptibility, and additional research is required. The use of enhanced susceptibility macaque models would benefit multiple facets of the HIV research field, including basic acquisition and pathogenesis studies as well as the vaccine and other biomedical preventions pipeline.

## Introduction

The use of nonhuman primates has proven to be a critical component of modeling and understanding mechanisms of HIV acquisition as well as testing biomedical interventions to prevent infection (reviewed in [1–5]). Macaque models have helped to describe mechanics of mucosal virus acquisition [2, 6, 7] and provide knowledge used to formulate effective prevention strategies. However, most macaque models of HIV acquisition used to develop and test biomedical HIV preventions utilize carefully controlled study arms and do not incorporate confounding experimental factors that could alter susceptibility, or infection risk. It is important to understand and consider these susceptibility-altering factors. A multitude of epidemiologic studies have highlighted coinfections, pre-existing conditions, certain behaviors, exogenous hormone use, and other factors widely found in at-risk human populations that are known or suspected to increase HIV infection risk [8–16]. In attempts to refine preclinical testing models, one could model susceptibility enhancement. Candidate preventions could then be evaluated with these models to ask whether the intervention is sufficiently protective to overcome the increased susceptibility and still prevent infection. However, macaque models of enhanced susceptibility models are few, and the number of models which could then be used to rigorously test biomedical interventions in such “real-world” contexts, and possibly better inform preclinical evaluations of HIV preventions, is also limited. Moreover, such models would have useful applications apart from prevention testing, including evaluation of suspected harmful products (e.g., those hypothesized to increase HIV acquisition risk based on epidemiologic observations, *in vitro* testing, etc.). Potentially, the use of animal models of increased susceptibility could be used to prevent failures in large-scale human clinical trials. There have been instances, particularly in HIV vaccine research, in which susceptibility to infection is unintentionally increased (e.g., HVTN 505, STEP Trial; [17, 18]). Thus, another benefit of an appropriate risk model could be to serve as an additional “gate keeper” of the safety of pre-clinical intervention candidates prior to advancement to clinical trials.

The paucity of risk models in macaques is a testament to the complex process of their development and study design validation. Our group has explored several experimental approaches for macaque models of enhanced susceptibility to HIV. In this review, we discuss our and others’ approaches to the development of such models and provide strategic considerations for designing macaque susceptibility models. Focusing primarily on modifiable risk factors (as opposed to internal host factors), this review discusses nonhuman primate models of enhanced HIV susceptibility using SIV (simian immunodeficiency virus) or SHIV (simian-HIV, chimeric virus of SIV expressing HIV envelope proteins), while highlighting their scarcity and the developmental difficulties. This review is organized such that reported findings and nonhuman primate models are discussed in the context of the studies’ analytical and/or design approaches. For each approach, we review the various susceptibility factors studied, models, and scientific findings and then highlight areas on which future studies might build upon and contribute to that model system of enhancement or increased infection risk or susceptibility (e.g., the ability to observe increased infections in experimental animals versus controls).

### Study design and statistical analysis approaches for susceptibility studies using nonhuman primate models

**Fig. 1:**
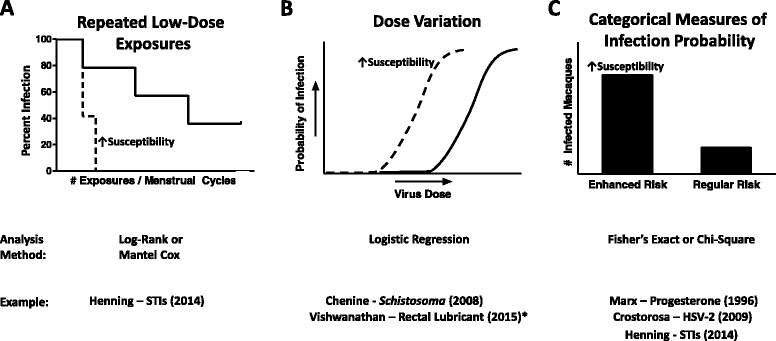
Schematic Designs of Enhanced HIV Susceptibility Nonhuman Primate Models. Three potential study designs and analytic approaches for macaque models of enhanced susceptibility to HIV infection. In panels a and b, the dotted line depicts the experimental arm, subjected to the susceptibility enhancement factor tested in the study. The solid line depicts the control arm. When increased susceptibility is observed, a left-shift would occur in the plots along the x-axis. Note that in the absence of observed susceptibility enhancement, the graphical depiction of the experimental arm would be similar to that of the control arm. **a** Repeated low-dose exposure infection rates are depicted in Kaplan-Meier survival curves and analyzed by log-rank testing. Survival, or confirmed macaque infections, are plotted on the y-axis, relative to either the number of virus exposures or, in our experience with cycling pigtail macaques, menstrual cycles during exposures. **b** Dose variation models evaluate the probability of infection (y-axis) relative to decreasing virus dose dilutions (or increasing concentration of challenge virus dose; x-axis). Animal infectious doses required to infect 50 % of the cohorts (AID_50_) are determined and analyzed by logistic regression statistical models. (*) Vishwanathan, et al. demonstrated lubricant-induced cytotoxicity, but not enhancement of SHIV acquisition risk. **c** Multiple study designs can use categorical measures of infection probability, such as Fisher’s exact or Chi-square outcome measures to analyze infection rates with and without the proposed enhancement factor

The design of a susceptibility or risk enhancement study to date has generally followed one of three approaches, or perhaps even a combination thereof: repeated exposures, analyzed by a log-rank test; dose titration comparison, commonly analyzed by a form of logistic regression; or presence/absence of infection (following exposure to the proposed enhancement factor), commonly analyzed by a probability statistic, such as a chi-square or Fisher’s exact test (Fig. 1). There does not seem to be a specific or best-suited strategy for any given type or group of susceptibility enhancement factors. For example, as will be further discussed below, modeling of risk enhancement with a coinfection (e.g., malaria, schistosomiasis, HSV-2, *Chlamydia trachomatis*, *Trichomonas vaginalis*) can be achieved with any of the three aforementioned analytical approaches [19–23].

### Repeated low-dose exposures

With the repeated exposures approach, a Kaplan-Meier plot (Fig. 1a) is usually constructed with the number of exposures along the x-axis, or, with naturally cycling female pigtail macaques [23], the number of menstrual cycles during exposures. Increased susceptibility is inferred by a significant left-shifted survival curve of the experimental arm (indicated by the dotted line in Fig. 1a), relative to the control curve. Using this approach, we recently described a pigtail macaque model of SHIV acquisition risk enhancement due to sexually transmitted infection (STI) coinfection with *Chlamydia trachomatis* and *Trichomonas vaginalis* [23] (Table 1). The susceptibility study compared infection rates between nine STI-positive and seven control macaques receiving repeated low-dose SHIV_SF162p3_ exposures_._ Because of the varying natural susceptibility during the menstrual cycle (pigtail macaques have lunar cycles, similar to humans) [24, 25], infections were analyzed by completed menstrual cycles (one menstrual cycle deemed equivalent to one SHIV susceptibility period) [23]. SHIV susceptibility was enhanced in STI-positive macaques (*p* = 0.04, log-rank), with a 2.5 times as high relative risk of infection, compared to controls (95 % CI 1.1, 5.6) [23]. A contributing factor to this enhancement is likely the increased levels of inflammatory mucosal cytokines observed during the STI inoculation and SHIV challenge phases [23].

**Table 1:** Literature Review of Macaque Models of Enhanced HIV Risk

Studied factor of susceptibility enhancement	Observed ↑susceptibility/infection risk	Discussion of enhancement/mechanism or potential susceptibility factor	Macaque gender/species	Virus stock/challenge dose	Study design parameters	References
Behavioral Factors of Susceptibility Enhancement						
Chronic Alcohol Use	No	Multiple, potential susceptibility factors: shifts in genital flora, increases in memory CD4+ T cells (viral targets), decreases in CD8+ T cells (anti-viral); ↑viremia in treated animals	Male and Female Rhesus	n/a	Designs vary; alcohol steadily administered via jacketed device	Loganantharaj (2014) [40]^a^
						Poonia (2006; AIDS)^a^[22]
						Poonia ([41]; JAIDS) (2006)^a^
Rectal Lubricant Use	No	Acute cytotoxicity observed after application of tested lubricant, but no ↑in risk during challenge phase	Male and Female Cynomologus	SHIV_SF162p3_ (varying doses)	Intrarectal challenge route; AID_50_ dose titration model	Vishwanathan (2015) [27]
Coinfections as Factor of Susceptibility Enhancement						
*C. trachomatis*/*T. vaginal* Coinfection (Genital Tract Infection)	Yes	2.5-fold ↑ risk in STI-positive animals; STI-positive animals infected in fewer menstrual cycles, compared to controls (*p* = 0.04, log-rank; *p* =0.02, Fisher’s exact). Increases in mucosal pro-inflammatory cytokines during STI inoculation and SHIV challenge periods	Female Pigtail	SHIV_SF162p3_ (10 TCID_50_)	Intravaginal challenge route; repeat low-dose exposures; exposures per menstrual cycle for risk assessment	Henning (2014) [23]
HSV-2 Coinfection (Genital Tract Infection)	Yes	Transmission not linked to active lesions. Possible HSV-2-induced immunosuppression impairs anti-SHIV response; subsequent study^b^ reported HSV-2 increases concentration of α4β7^high^ CD4+ T cells (viral targets)	Female Rhesus	SHIV-RT (200 and 10^3^ TCID_50_) ↑Risk with 10^3^	Intravaginal challenge route; animals either treated 1× or 2× with DMPA, then either 200 or 10^3^ TCID_50_	Crostarosa (2009) [22]
						Martinelli [36]^b^ (2011)
Malaria (Systemic Infection)	No	Evidence of potential susceptibility factors ;↑ viral load and CCR5+ CD4+ T cells (viral targets) in *P. fragile*-infected animals, but risk/hazard ratio not determined	Male Rhesus	SIVmac239 (10^3^ TCID_50_)	Intravenous challenge route, comparing control and coinfected groups	Trott (2011) [48]^a^
Schistosomiasis (Helminth/Systemic Infection)	Yes	17-fold lower dose of virus required to infect *S. mansoni*-infected animals; ↑ viremia and replication in CD4+ central memory cells (viral targets)	Female Rhesus	SHIV-1157ipd3N4 (Clade C; varying doses)	Intrarectal challenge route; AID_50_ dose titration model	Chenine (2008) [21]
Schistosomiasis (Helminth/Systemic Infection)	No	Intravenous challenges (compared to mucosal challenges) did not result in same increase of SHIV acquisition risk	Female Rhesus	SHIV-1157ipd3N4 (Clade C; varying doses)	Intravenous challenge route; AID_50_ dose titration model	Siddappa (2011) [30]
Hormonal Factors of Susceptibility Enhancement						
Hormone Levels Associated with Menstrual Cycle Phase (Endogenous Hormone)	Yes^c^	Exact mechanisms to be determined. Increased rates of SHIV RNA detection in late-luteal and menses phases	Female Pigtail	SHIV_SF162p3_ (50 TCID_50_)	Intravaginal challenge route; repeat low-dose exposures	Vishwanathan (2011) [25]^a^
						Kersh (2014) [24]^a^
Phase of Menstrual Cycle	No	Vaginal application of cell-free virus resulted in infection; 50 % of macaques infected in luteal phase, compared to 24 % challenged in follicular phase (not statistically significant)	Female Rhesus	SIVmac251 (3 × 10^1^ to 3 × 10^3^ TCID_50_, cell free; 2 to 1 × 10^4^ infected PBMCs, cell-associated)	Compared infectivity of different doses of cell-free vs. cell-associated virus via intravaginal vs. intravenous inoculation routes	Sodora (1998) [39]^d^
Progesterone Implants (Exogenous Hormone)	Yes	7.7-fold ↑ risk in implanted animals. DMPA induced significant vaginal thinning, with ↑peak and 1st 3 months of viremia	Female Rhesus	SIVmac251 (640 TCID_50_)	Single intravaginal challenge with determined “minimal vaginal dose”	Marx (1996) [35]
Vaccines as a Factor of Susceptibility Enhancement						
Ad5^d^ Vaccine (Vaccine-induced enhancement)	Yes	↑ risk in Ad5 seropositive animals infected with the lower (10^3^ TCID_50_) challenge dose; study recapitulates lack of Ad5 vaccine efficacy and model vaccine-induced acquisition risk enhancement	Male Rhesus	SIVmac251 (varying doses)	Penile challenge route; 10-fold increases in virus concentration (10^3^ to 10^5^); comparisons among groups +/− Ad5 immunity, SIV vaccination, and naïve controls	Qureshi (2012) [31]

Relevant literature is grouped by type of susceptibility enhancement factor, with description of study design, parameters, and analytical approach and effect(s) of enhancement factor(s)

Terminology of ‘increased infections in experimental animals over controls’ and ‘increased (↑) risk/susceptibility’ is synonymous with observed enhancement of SIV/SHIV infection susceptibility due to or attributed to the studied (potential) enhancement factor

^a^Citations describe studies not reporting or not designed to demonstrate enhanced susceptibility, but describe potential or key susceptibility factors for HIV/SIV/SHIV infection

^b^Follow-up study to Crostarosa, et al. [22] publication, investigating mechanisms of HSV-2-induced enhanced susceptibility

^c^Assessed retrospectively, during specific phases of the menstrual cycle

^d^Ad5 – Adenovirus, serotype 5

With a repeated low-dose exposure susceptibility model, it is important to acknowledge the difficulty in determining the appropriate low virus dose when developing these models, and it is critical to identify the optimal virus dose that leaves sufficient numbers of control animals uninfected after repeated challenges. Depending on the extent of virus stock characterization (and considering also the route of challenge), determining this ideal challenge dose may require many macaques for virus titration studies prior to a susceptibility study.

### Dose variation

Another option would be to compare virus dose titrations between experimental groups (e.g., with and without hypothesized enhancement factor; Fig. 1b). Using this study design, animals are first challenged with very dilute virus, and, after confirming the absence of infection, challenges continue in similar fashion with increasing concentrations of virus until an animal is infected. A benefit to this design is that, after resting, uninfected animals can be re-enrolled. However, a potential problem is that upon re-challenge of exposed animals, naturally more resistant animals may accumulate and confound results. The virus dose required to infect 50 % of the animals (AID_50_) is calculated for each experimental group and statistically compared, often using a form of logistic regression analysis (described in detail by Spouge and subsequently used or adapted in other studies [21, 26, 27]). Chenine, et al. used this AID_50_ study design to model and demonstrate enhanced susceptibility to SHIV infection due to systemic helminth coinfection with *Schistosoma mansoni* [21]. Chenine, et al. [28] and Ayash-Raskovsky, et al. [20] first alluded to potential risk enhancement by reporting that schistosomiasis both reactivates SHIV and increases viral replication rates. The subsequent susceptibility study in rhesus macaques used intrarectal challenges with varying doses of a SHIV Clade C infectious molecular clone [29] (Table 1). The infecting virus dose was 17-fold lower in *S. mansoni*-infected animals, compared to controls [21] (*p* < 0.001, logistic regression methods of Spouge [26]). The authors described possible mechanisms for increased susceptibility by showing higher concentrations of viral target cells in *S. mansoni*-infected macaques and higher levels of viral replication in these cellular subsets [21]. To address whether the increased risk was due to mucosal inflammation and/or subsequent target cell recruitment, or if systemic effects of schistosomiasis increased SHIV infection risk, a follow-up risk study was performed again using a virus titration study design, except, in this study, macaques were challenged intravenously [30] (Table 1). In this subsequent study, the AID_50_ and peak virus level between *S. mansoni*-infected and control groups were not significantly different, suggesting susceptibility is facilitated by the helminth’s interaction at the mucosa, resultant inflammation, and/or the consequent upregulation of viral replication at the mucosal surfaces [30].

In an HIV vaccine model using rhesus macaques, Qureshi, et al. also used a dose escalation model [31] (Table 1) to determine if a nonhuman primate model could recapitulate the enhanced susceptibility to HIV infection seen in the human phase III STEP trial [17, 31]. In the trial, preexisting seropositivity to adenovirus serotype 5 (Ad5) was associated with increased HIV-1 infections in men who received an Ad5 HIV vaccine. In the macaque model, escalating doses of SIVmac251 were given through repeated penile exposures to animals infected with adenovirus and then vaccinated with an Ad5 SIV vaccine [31]. In this model, an enhanced SIV infection rate was observed in animals with pre-existing Ad5 immunity that received low doses (10^3^ TCID _50_) of SIV, but not higher doses. The analytic approaches supporting this finding included log-rank tests of survival, determination of relative risks of infection and likelihood-ratios in statistical models with an assumption of a leaky vaccine effect. Moreover, when examining the difference in the number of infections in the nonhuman primate and human models, the enhancement was marginal in both, but of a similar order of magnitude (two in 43 macaques, compared to 16 in approximately 1800 men). These important findings demonstrate that macaque models can be used to determine if biomedical preventions increase susceptibility to SIV.

Our group has also employed the use of virus dose titration challenges to evaluate the effect of rectal lubricants on rectal SHIV acquisition risk [27] (Table 1). This study was first conceptualized after recent *in vitro* and *ex vivo* studies reported detrimental effects of lubricants on rectal and genital epithelium [32–34]. The cytotoxicity phase of the study showed acute effects of a highly hyperosmolar lubricant on macaque anorectal tissues [27]. Because of the overt susceptibility factors (local inflammatory response, rectal bleeding, epithelial sloughing), a susceptibility phase was conducted employing the AID_50_ study design. Despite the acute cytotoxicity, the difference in SHIV_SF162p3_ doses required to infect controls versus lubricant-treated animals was not statistically different. While this particular study did not report increased risk, it is prudent to note that despite plausible mechanistic data, models may not identify increased susceptibility or be sufficiently sensitive to demonstrate modest increases in susceptibility. Vishwanathan, et al. discussed that possibly the acute nature or type of induced inflammation was not sufficient to increase infection risk to levels above the 2-fold theoretical threshold limit with the virus dose-titration challenge model, or perhaps the highly regenerative nature of the rectal mucosa negates inflammatory effects of lubricant application [27]. Of note, follow-up studies in rhesus macaques will use a repeat low-dose design (instead of AID_50_) as an alternate model to further evaluate possible risk enhancement due to rectal lubricant use.

### Categorical measures of infection probability

The use of a probability analysis, such chi-square or Fisher’s exact measures (Fig. 1c) can be applied to a variety of study designs, including repeat low-dose and dose titration models. This direct comparison considers the relationship between infection (with the challenge virus) and the nominal, or categorical, variables of the presence/absence of the proposed enhancement factor. Fisher’s exact test is often more appropriate than a chi-square analysis because of the smaller sample sizes necessitated in nonhuman primate research. As with the repeated exposure model, selecting the appropriate virus dose is also an important factor when designing susceptibility challenge studies with Fisher’s exact analyses. The use of too high a dose will result in all animals becoming infected and loss of the ability to observe enhancement between experimental groups.

While studying the effects of progesterone implants in macaques, Marx, et. al, provided one of the first descriptions of Depo-Provera’s, or depot medroxyprogesterone acetate (DMPA), effect on susceptibility to SIV acquisition and used Fisher’s exact analyses to demonstrate increased SIV risk due to exogenous hormone use [35] (Table 1). In this study, using an analytical approach depicted in Fig. 1c, female rhesus macaques received subcutaneous implants of progesterone-containing pellets, while the control group received placebo implants. After vaginal challenge with SIVmac251, cell-associated SIV was detected in 78 % of DMPA-treated animals, but only 10 % of controls (*p* < 0.008, Fisher’s exact), 7.7 times the risk [35].

Crostarosa, et al. developed a vaginal herpes simplex virus type-2 (HSV-2) coinfection model in female rhesus macaques and used the model to evaluate susceptibility to vaginal infection with SHIV-RT [22] (Table 1). They employed a complex study design which also factored the role of DMPA on susceptibility, though direct or statistically significant conclusions were not made on the effect of the exogenous hormone on susceptibility. Overall, using an approach similar to Fig. 1c, conclusions were drawn from *n* = 28 macaques and *n* = 46 macaque infections/challenges in which macaques remaining SHIV-RT-negative were reused in subsequent arms. HSV-2-positive animals which received only one hormone injection showed increased susceptibility to SHIV-RT infection at 10^3^ TCID_50_ (100 % infection), relative to HSV-2-negative controls which also received one Depo-Provera dose (46 % infection) (*p* < 0.05, Fisher’s exact) [22]. This model was then used to elucidate mechanisms of the enhanced susceptibility, such as increased dendritic cell availability as a viral target cell and immunomodulation of viral replication [36], and to provide a more stringent model system for evaluating efficacy of non-nucleoside reverse transcriptase inhibitor (NNRTI)-containing microbicides [22, 37] (Table 1).

In our *C. trachomatis*-*T. vaginalis* coinfection study, we corroborated log-rank analyses of enhanced risk with Fisher’s exact testing [23]. By evaluating the number of menstrual cycles with and without SHIV infection among the two experimental groups, STI coinfection was once again associated with increased risk of vaginal HIV acquisition (*p* = 0.02; [23]) (Table 1). This report is an example of how a multi-statistic approach can be used to analyze (and potentially strengthen) observations of enhanced susceptibility.

### Observations on infection susceptibility from varying study designs

While the focus of this review has been on research using nonhuman primate models of HIV susceptibility, it is still prudent to acknowledge the wealth of other studies in macaques providing evidence of important and relevant susceptibility factors, albeit not in the framework of a susceptibility-virus challenge study. For example, it has been speculated that HIV susceptibility varies throughout the menstrual cycle in humans [38], as demonstrated in macaques challenged with S(H)IV [24, 25, 39]. Sodora, et al. first described the propensity for greater rates of infection in the luteal versus follicular phase of the menstrual cycle in rhesus macaques [39]. Our group addressed the same topic through retrospective studies in female pigtail macaques by analyzing time points of first plasma SHIV_SF162p3_ RNA detection from repeat low-dose intravaginal challenge studies relative to the phase of the menstrual cycle [24, 25] (Table 1). The majority of infections were detected in the follicular phase, with imputed infection dates (after correcting for a viral eclipse period) in either the menstrual or pre-menstrual phases [24, 25]. While these studies were not prospectively designed to model increased risk per se [24, 25, 39], they demonstrate varying and enhanced susceptibility to S(H)IV infection during certain phases of the menstrual cycle.

Evidence of susceptibility factors relating to modifiable behavioral risk factors have also been reported from macaque models of chronic alcohol consumption. Chronic alcohol usage induces adverse shifts in cellular and microbial populations in the genital tract, increases concentrations of viral target cells, gut memory CD4+ T cells, reduces levels of cytolytic T cells, and, in SIV-infected animals, was associated with higher levels of viremia [40–42] (Table 1). From human studies, there is empirical evidence for the likelihood of HIV risk enhancement by other substances, such as cocaine; however, macaque models have not yet been developed to further investigate this factor [43]. Related to alcohol consumption, further exploration of susceptibility models which also evaluate the role of gut T regulatory and Th17 cells would benefit the field. As these and other CD4+ cell populations are known to mitigate host response to the virus, control viral activation and spread, and play a key role in disease progression, macaque susceptibility enhancement models incorporating the further study of their role in resistance and susceptibility to infection could prove critical for HIV prevention efforts [44–47].

In addition to helminth infections, other systemic infections, such as malaria, have been modeled in nonhuman primates through which evidence of increased SIV risk was observed. Rhesus macaques coinfected with *Plasmodium fragile* had significantly higher ramp-up levels of plasma viremia and increased concentrations of CCR5 + CD4+ T cells (SIV target cells). The study showed evidence of increased susceptibility but did not specifically evaluate relative risk or hazard ratios [48] (Table 1). Studies such as these provide impetus for the development of additional nonhuman primate susceptibility models and execution of susceptibility-virus challenge studies designed to examine the effect of these factors on virus acquisition, potentially using an approach discussed in this review.

## Discussion

This review discusses study design strategies and reported models of enhanced susceptibility using the framework of three statistical approaches. The field of nonhuman primate models for HIV acquisition, pathogenesis, and efficacy testing of prevention methods is comprehensive, with detailed literature and a great depth of understanding of how these models can be applied and how the generated data should be interpreted. In comparison, there is a paucity of nonhuman primate models for assessing susceptibility to HIV (using SIV/SHIV) and for evaluating factors that might increase the risk of virus acquisition. And these relatively few studies also underscore the difficulty in developing efficient (e.g., small animal numbers, streamlined design), relevant (e.g., effect size, applicability to the epidemic), and reliable (e.g., low *p* values, narrow confidence intervals) nonhuman primate model systems to examine increased susceptibility. Developing and establishing such studies may require large numbers of animals, with multiple study arms to fine-tune study design (for example, the Crostarosa, et al. study used a total of 28 macaques [22] and Qureshi, et al. used 43). The studies described here also provide insight into factors enhancing, or with the potential to enhance, susceptibility to HIV (or SIV/SHIV) infection. When designing models of enhanced susceptibility, one is also faced with the appropriate selection of relevant factors or a reliable study design approach to best represent the epidemic. For example, what is the most relevant genital tract pathogen or form of hormonal contraception to use? Even the basic question of virus stock selection can have great bearing on study outcome, relevance to the epidemic, and the nature of the experimental question(s) [49]. The repeat low-dose model has been successfully used in efficacy studies [50–54], but perhaps a virus dose-titration (AID_50_) model might provide greater power to detect increases in risk. The ability to anticipate the magnitude of risk from epidemiologic or *in vitro* studies could inform model selection. The log-rank or dose escalation approach may provide greater power in evaluating acquisition risk in the lower ranges of magnitude, or modest risk enhancement, whereas Fisher’s exact testing is perhaps more appropriate in analyzing higher magnitudes of susceptibility enhancement. It is possible that the most relevant and robust model system utilizes a combination of study design approaches described herein. For example, Qureshi, et al. elegantly described the use of multiple analytical strategies in their Ad5 vaccine enhancement study [31]. Even when acquisition risk is anticipated as marginal, it is possible that the appropriate selection of challenge route, virus titer and statistical approach can still capture the extent of the risk enhancement.

We’ve highlighted three possible, and previously described [21, 23, 27, 31, 35] study design and statistical analytical approaches, but readily acknowledge that this field of research is limited and that future models and study designs could, and perhaps should, be developed to better address the question of increased HIV susceptibility. It is possible that a novel system altogether would be most appropriate for conducting susceptibility studies. Ideally, a side-by-side comparison of these varying study design approaches would be conducted, testing the performance of a factor known to enhance infection risk (e.g. STIs, DMPA) in each model system. Such an experiment, albeit resource intensive, would provide a well-controlled comparison of the various modeling strategies and statistical analyses.

Nonhuman primate studies incorporating enhanced susceptibility models are capable of supporting epidemiologic findings of HIV risk, defining susceptibility mechanisms or factors, and identifying targets for focused interventions. Moreover, models of enhanced susceptibility provide a system with which biomedical preventions can be rigorously tested in the context of “real-world” susceptibility conditions, prior to proceeding to complex and costly human clinical trials. As previously mentioned, the use of an increased risk model to test candidate clinical trial prevention strategies could alert us to possible susceptibility enhancement, prior to the introduction in humans. One could argue the paucity of such models is a hindrance to the advancement of broadly efficacious HIV prevention strategies. The field of HIV prevention research still doesn’t have the “perfect” model of HIV acquisition, a goal that may indeed be unattainable. Nonetheless, efforts to refine our models may necessitate a combination of varying study designs for differing genders, infection routes and cofactors. Continued work in the development and use of nonhuman primate models of enhanced susceptibility could, or would, increase our understanding of acquisition and provide better models for prevention testing.

## Abbreviations

Ad5: Adenovirus, serotype 5
AID_50_: Animal infectious dose required to infect 50 % of cohort
DMPA: Depot medroxyprogesterone acetate
HIV: Human immunodeficiency virus
HSV-2: Herpes simplex virus, type 2
HVTN-005: HIV (human immunodeficiency virus) vaccine trials network, clinical trial number 505
NNRTI: Non-nucleoside reverse transcriptase inhibitor
PBMC: Peripheral blood mononuclear cells
RNA: Ribonucleic acid
SHIV: Simian-human (chimeric) immunodeficiency virus
SIV: Simian immunodeficiency virus
SHIV-RT: Simian-human (chimeric) immunodeficiency virus, expressing human immunodeficiency virus reverse transcriptase enzyme
STEP (trial): Not an acronym; represents a Merck-sponsored human immunodeficiency virus vaccine trial, also known as HVTN (human immunodeficiency virus vaccine trials network) 502
STI: Sexually transmitted infection(s)
TCID_50_: Tissue culture infectious dose required to infection 50 % of tested samples/cohort
